# Overexpression of Programmed Cell Death 1 Prevents Doxorubicin-Induced Apoptosis Through Autophagy Induction in H9c2 Cardiomyocytes

**DOI:** 10.1007/s12012-022-09726-w

**Published:** 2022-02-21

**Authors:** Syu-ichi Kanno, Akiyoshi Hara

**Affiliations:** Department of Clinical Pharmacotherapeutics, Tohoku Medical and Pharmaceutical University, 4-4-1 Komatsushima, Aoba-ku, Sendai, 981-8558 Japan

**Keywords:** Doxorubicin, Cardiomyocyte, Cardiotoxicity, Programed cell death 1, Autophagy, Apoptosis

## Abstract

Doxorubicin (DOX) is a potent chemotherapeutic agent; however, it causes severe heart injury via apoptosis induction in many patients. DOX-induced cardiotoxicity is attenuated by activated autophagy in the heart. We previously found that programmed cell death 1 (Pdcd1), an immune checkpoint receptor, inhibits DOX-induced cardiomyocyte apoptosis. In this study, we investigated whether autophagy contributes to the protective role of Pdcd1 against DOX-induced cardiomyocyte apoptosis. We also examined the role of Pdcd1 in DOX-induced apoptosis in cancer cells. Rat cardiomyocyte cell line H9c2 and human cancer cell lines K562 and MCF-7 were transfected with Pdcd1-encoding plasmid DNA to establish Pdcd1-overexpressing cells. Apoptosis and autophagy were determined using a luciferase assay. In H9c2 cells, DOX-induced apoptosis and viability reduction occurred through caspase activation. In particular, Pdcd1 overexpression activated the autophagy pathway through the inhibition of the mammalian target of rapamycin, a major negative regulator of autophagy. Moreover, it prevented DOX-induced cardiomyocyte apoptosis; a similar cardioprotection was observed when normal H9c2 cells (without Pdcd1 overexpression) were treated with rapamycin, an autophagy inducer, before the DOX treatment. Conversely, in cancer cells, Pdcd1 overexpression increased both basal and DOX-induced apoptosis. The role of Pdcd1 in DOX-induced apoptosis in cardiomyocytes and cancer cells was opposing. Pdcd1 signaling prevented DOX-induced apoptosis in cardiomyocytes, through autophagy induction; it enhanced DOX-induced apoptosis in cancer cells. Therefore, Pdcd1 could be a critical molecule for more effective and safer DOX chemotherapy.

**Supplementary Information:**

The online version contains supplementary material available at 10.1007/s12012-022-09726-w.

## Introduction

Doxorubicin (DOX), an anthracycline chemotherapeutic agent, is widely and effectively in the treatment of various solid and hematological malignancies [1, 2]. However, it is known to inflict severe heart damage, with cardiotoxicity being one of its most significant adverse effects [1–4]. The risk of heart failure may be augmented by pre-existing loading conditions, such as hypertension and valvular disease [5], and in combination with other chemotherapy drugs [6]. The prevention of adverse cardiotoxicity effects of DOX without the loss of its anti-tumor effects will provide effective and safe chemotherapy for various types of cancer. Therefore, several pharmacological approaches to reduce DOX cardiotoxicity and subsequent heart failure have been proposed in animal and human studies [4, 7, 8].

DOX cardiotoxicity is mediated by at least two major pathways of apoptosis and autophagy [4, 8–12]. Although DOX-induced apoptosis is an important process involved in the anti-tumor effects of DOX in cancer cells, it can be responsible for cardiotoxicity in cardiomyocyte cells [1, 4, 13]. Autophagy, a cellular housekeeping process, has dual functions in cell survival and death, depending on the cellular context [14, 15]. Interestingly, the crosstalk between autophagy and apoptosis in DOX-induced cardiotoxicity has been demonstrated in many recent studies. Several findings indicate that the activation of autophagy is effective in reducing DOX-induced cardiac apoptosis and toxicity [9, 16–19]. Several other findings suggest that autophagy contributes to the development or exacerbation of DOX-induced cardiac apoptosis [20–24]. For example, rapamycin (Rap), an inducer of autophagy, has been reported to prevent [17, 18] or promote [23] DOX-induced cardiac apoptosis. Similarly, preventive [24] or promotive [17] effects on DOX-induced apoptosis have also been observed with bafilomycin A1 (Baf), an inhibitor of autophagy. Thus, the role of autophagy in DOX-induced apoptosis is inconsistent between studies and remains unclear.

We have previously reported that the mRNA expression of programmed cell death 1 (Pdcd1 or PD-1) in blood is a predictive and protective factor for DOX-induced cardiotoxicity, and that the cardiomyocyte H9c2 cells in which Pdcd1 was knocked down showed a marked increase in apoptosis upon exposure to DOX [25]. Pdcd1 is a typical immune checkpoint molecule, and its monoclonal antibodies are used to treat different types of solid or hematological tumors [26–28]. According to recent clinical trials, a combination chemotherapy with DOX and nivolumab, a monoclonal antibody against Pdcd1, exhibited more effective anti-tumor activity in cancer patients [26, 28]. However, the inhibition of Pdcd1 activity can produce a wide spectrum of immune-related adverse events such as autoimmune myocarditis [29] and dilated cardiomyopathy [30].

Nevertheless, the molecular mechanisms underlying the cardioprotective action of Pdcd1 are not fully understood. The beneficial activity of Pdcd1 against DOX-induced apoptosis and toxicity could possibly be attributed to the activation or inhibition of autophagy. To test this hypothesis, we established the overexpression of Pdcd1 in H9c2 cells and investigated whether the autophagy pathway is involved in the mechanisms of the anti-apoptotic action of Pdcd1. We also examined the effects of Pdcd1 overexpression on the DOX-induced apoptosis of human cancer cell lines K562 and MCF-7.

## Materials and Methods

### Materials

DOX was purchased from Sandoz K. K. (Tokyo, Japan). Rapamycin (Rap) and bafilomycin A1 (Baf) were purchased from Wako Pure Chemical Industries, Ltd. (Osaka, Japan). Z-Asp-CH_2_-DCB (Z-Asp) was purchased from PEPTIDE Institute, Inc. (Osaka, Japan). The Pdcd1 overexpression plasmids were constructed by GeneArt™ Gene Synthesis (Thermo Fisher Scientific, Waltham, MA, USA). Primary antibody against Pdcd1 was purchased from Proteintech (Rosemont, IL, USA). Primary antibodies against light chain 3B (LC3B), mTOR, phosphorylated mTOR (p-mTOR), Beclin-1, autophagy gene 3 (Atg3), autophagy gene 5 (Atg5), caspase-3, Bcl-2-associated agonist of cell death protein (Bad), Bcl-2-associated X protein (Bax), β-actin, and secondary antibody (horseradish peroxidase-conjugated anti-rabbit immunoglobulin G) were purchased from Cell Signaling Technology (Danvers, MA, USA). Fluorescent secondary antibodies were purchased from Thermo Fisher Scientific. All other reagents, unless stated, were of the highest grade available and were purchased from Sigma Chemical Co. (St. Louis, MO, USA) or Wako Pure Chemical Industries, Ltd.

### Cell Culture

Rat embryonic cardiomyoblast-derived H9c2 (2–1) cell line was obtained from DS Pharma Biomedical (Osaka, Japan). The human breast cancer cell line MCF-7 and the human erythroleukemia cell line K562 were obtained from the Cell Resource Center for Biomedical Research, Tohoku University (Sendai, Japan). H9c2 cells were maintained in Dulbecco’s modified Eagle’s medium (DMEM) and K562 and MCF-7 cells were maintained in RPMI1640 supplemented with 10% fetal bovine serum, 100 U/mL penicillin G, and 100 μg/mL streptomycin at 37 °C in a humidified incubator with 5% CO_2_ and 95% air under standard conditions. Viable cell counts were determined by staining with 0.2% Trypan blue. To maintain exponential growth, cells were seeded at a density of 5 × 10^4^ cells/mL and passaged every 3–4 days. For the remaining assays, cells were cultured in 2-mL aliquots in 35-mm petri dishes.

### Cell Viability Assay

We examined the effects of Rap, Baf, and Z-Asp on the viability of DOX-treated H9c2 cells. Rap promotes the autophagy pathway by inhibiting mTOR, a major negative regulator of autophagy [31]. Baf inhibits the autophagy pathway by inhibiting the vacuolar H^+^-ATPase, thereby impairing autophagosome–lysosome fusion [32]. Z-Asp inhibits the apoptotic pathway by acting as a broad caspase inhibitor.

The relative cell viability was determined by a luminescence assay based on the adenosine triphosphate quantification assay (CellTiter-Glo® 2.0; Promega, Madison, WI, USA). Briefly, H9c2 cells were seeded in a 96-well white microplate and cultured overnight. Subsequently, the cells were incubated with 50 nM Rap, 10 nM Baf, 100 μM Z-Asp, or 0.1% dimethyl sulfoxide (control) for 1 h and then with 1 μM DOX for 18 h. The CellTiter-Glo® reagent was added, and luminescence was read on a Varioskan™ LUX multimode microplate reader (Thermo Fisher Scientific). Data were normalized to control cells and presented as percentages of viable cells.

### Transfection

The Pdcd1 overexpression plasmid was transfected into cells using ViaFect™ Transfection Reagent (Promega) according to the manufacturer’s protocol. Briefly, cells were seeded into 35-mm dishes (8 × 10^4^ cells) or 96-well microplates (4 × 10^3^ cells) in antibiotic-free medium and cultured overnight. The transfection complex was mixed with 1 µg of plasmid DNA and 3 µL of ViaFect™ Transfection Reagent and incubated for 10 min at room temperature. The cells were supplied with fresh medium and incubated with the transfection complex for 24 h in a 37 °C incubator. Thereafter, the Pdcd1-overexpressing cells or negative control (mock) cells were used for the experimental study.

### Apoptosis Assay

We assessed the induction of apoptosis using the RealTime-Glo™ Annexin V Apoptosis assay and the Caspase-Glo® 3/7 Assay (Promega) [25] or nuclear morphological observation [33] using our previously described method.

Briefly, the cells were distributed in a 96-well plate at a density of 4 × 10^3^ cells per well and allowed to adhere overnight. For the RealTime-Glo™ Annexin V Apoptosis assay, the cells were incubated with 1 μM DOX in the presence of Annexin V luciferase reagents, and time-dependent increases in luminescence reflecting the apoptotic process were monitored. Similarly, apoptosis was evaluated after incubation for the indicated times with DOX concentrations of 0.03, 0.1, 0.3, or 1 μM. For the Caspase-Glo® 3/7 assay, the cells were incubated with DOX (0.03–1 μM) for the indicated times. The Caspase-Glo® 3/7 reagents were added to each well, and the contents were gently mixed. The resulting luminescence intensity was measured using a Varioskan™ LUX multimode microplate reader (Thermo Fisher Scientific). The results were calculated as the percentage of control groups and expressed as relative changes.

Nuclear morphological changes were assessed using the fluorescence reagent bisbenzimide Hoechst 33342 fluorochrome trihydrochloride (H33342). After incubation with DOX for 24 h, the cells were stained for 10 min at room temperature in phosphate-buffered saline (PBS) containing 5 μM H33342 and observed under a model C-1 fluorescence microscope (Nikon, Tokyo, Japan) using the excitation and emission filters of 360 and 420 nm, respectively. Apoptosis was characterized by chromatin condensation followed by partitioning into multiple bodies. At least 300 cells were counted in each experiment, and the percentage of apoptotic cells was calculated.

### Caspase-3/7 Activity Detection Assay

The caspase-3/7 activity was assessed using the Caspase-Glo® 3/7 Assay (Promega), which involves incubation with a luminogenic caspase-3/7 substrate, followed by caspase cleavage of the substrate and generation of a luminescent signal. The cells were seeded on a 96-well white culture plate at a density of 4 × 10^3^ cells per well and cultured overnight. The cell culture medium containing various concentrations of DOX was added to H9c2 (0.1, 0.3, or 1 μM) and K562 cells (0.03, 0.1, 0.3, or 1 μM) for the indicated times. The Caspase-Glo® 3/7 reagents were added to each well, and the contents were gently mixed. The luminescence of each sample was measured using a Varioskan™ LUX multimode microplate reader (Thermo Fisher Scientific), and the results are expressed as a percentage relative to the control group.

### Autophagy Assay

Autophagy was evaluated using the Autophagy LC3 HiBiT Reporter Vector and Detection System (Promega) or DAL Green-Autophagy Detection reagent (Dojindo, Kumamoto, Japan), according to the manufacturer’s instructions.

We stably expressed the autophagy LC3 HiBiT reporter in H9c2 cells (H9c2/LC3) by transfecting them with the LC3 HiBiT reporter plasmid vector. For cell maintenance and propagation of a sequence encoding the LC3 reporter gene in cells, the cells were selected with the cell culture medium containing 800 µg/mL G418 antibiotic. The H9c2/LC3 cells were plated into a 96-well white microplate and incubated with the drugs for each indicated time. Subsequently, the Nano-Glo® HiBiT Lytic Reagent (Promega) was added and luminescence was measured, with altered assay signals reflecting changes in autophagic flux and related reporter degradation. The induction of autophagy results in decreased reporter levels and luminescent signal, while its inhibition results in increased reporter levels and luminescent signal. The reporter levels of the treated cells were expressed as percentages relative to the corresponding control cells.

We used the DAL Green assay, which is a simple and direct approach for detecting autophagy in cells; it employs a fluorescent autolysosome marker. After transfection with Pdcd1, the medium was removed, and the cells were incubated with 1 μM DAL Green reagent at 37 °C with 5% CO_2_ for 1 h. Autolysosome cells were observed by fluorescence microscopy.

In addition, we confirmed the detection of LC3B, an autophagy marker, by immunofluorescence as follows.

### Immunofluorescence

Cells were seeded into Lab-Tek® 8-well chambered cover glass system plates (Thermo Fisher Scientific) at 4 × 10^4^ cells/mL and incubated overnight in antibiotic-free medium. Cells were transfected with the Pdcd1overexpression plasmid and cultured for 24 h. The chambered slides were washed twice with PBS adjusted to pH 7.4 and fixed in ice-cold 1:1 methanol:acetone for 30 min. The slides were immersed for 10 min in 1% goat serum and 0.25% Triton X-100 in PBS and then transferred to the Blocking One Histo reagent (Nacalai Tesque, Kyoto, Japan) for 10 min. The slides were washed with PBS containing 0.1% Tween 20, incubated with primary antibodies: anti-Pdcd1 mouse monoclonal antibody (diluted 1:250; cat. no. 66220–1-Ig, Proteintech), anti-LC3B rabbit monoclonal antibody (diluted 1:10,000; cat. no. 43566, Cell Signaling Technology), or anti-p-mTOR rabbit monoclonal antibody (diluted 1:100; cat. no. 5536, Cell Signaling Technology) for 1 h at room temperature, washed with PBS, and then incubated with respective anti-mouse or anti-rabbit Alexa Fluor-conjugated secondary antibodies (diluted both 1:1000; cat. no. A32742 and cat. no. A32740, respectively; Thermo Fisher Scientific) for 1 h. After rinsing with PBS, a drop of Fluoro-KEEPER Antifade Reagent (Nacalai Tesque) was added to each well. Cells were observed under a fluorescence microscope, and cell components were visualized based on the fluorescence intensity in blue (405 nm) for nuclear DNA and red (594 nm) for each target protein-positive cells.

### Western Blot

The cells were washed with PBS and lysed in CelLytic M® (Sigma) to collect the total cell lysate, according to the manufacturer's instructions. Protein concentration was measured using the BCA™ Protein Assay Kit (Thermo Fisher Scientific) according to the manufacturer’s instructions. Following electrophoresis of protein samples (10 μg) on a 5%–15% SDS–polyacrylamide gel, the proteins was transferred to a polyvinylidene difluoride (PVDF) membrane, which was subsequently blocked with Blocking One® (Nacalai Tesque) for 1 h and then incubated with primary antibodies: anti-mTOR, anti-p-mTOR, anti-Beclin-1, anti-Atg3, anti-Atg5, anti-Caspase-3, anti-Bad, anti-Bax, anti-β-actin (all diluted 1:1000; cat. nos. 2983, 5536, 3738, 3415, 12,994, 9662, 9292, 2772, 4967, Cell Signaling Technology), or anti-Pdcd1 (1:2000; cat. no. no. 66220-1-Ig, Proteintech), overnight at 4 °C. The membrane was then washed with wash buffer (PBS containing 0.05% Tween-20) and incubated with anti-rabbit conjugate horseradish peroxidase-linked secondary antibody (diluted 1:5000; cat no. 7074, Cell Signaling Technology) for 1 h. Protein expression was determined using enhanced chemiluminescence with the Pierce® Western Blotting Substrate (Thermo Fisher Scientific) and chemiluminescence image detection system (LAS-4000, GE Healthcare, Tokyo, Japan).

### Statistical Analysis

Each experiment was repeated three times, and the results were presented as the mean ± standard error of the mean (SEM). Differences between the two groups were compared using one-way analysis of variance (ANOVA). Multiple comparisons were performed using one-way ANOVA followed by Tukey’s post hoc test. *p* values less than 0.05 were considered statistically significant. Statistical analyses were performed using the BellCurve for Excel software (Social Survey Research Information Co., Ltd., Tokyo, Japan).

## Results

### Effects of Rap, Baf, or Z-Asp on Apoptosis, Autophagy, and Cell Viability in DOX-Treated H9c2 Cells

**Fig. 1:**
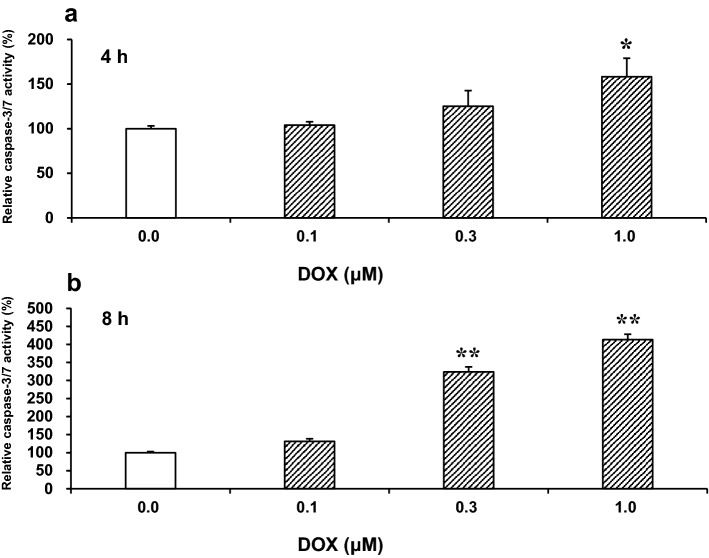
Doxorubicin (DOX)-induced changes in caspase-3/7 activity in H9c2 cells. The cells treated with various concentrations of DOX (0.1–1 μM) for **a** 4 h or **b** 8 h were assessed for their caspase-3/7 activity, using a luciferase assay. The degree of caspase-3/7 activity was expressed as a percentage of untreated control cells. Data are presented as the mean ± SEM of three samples. *p < 0.05, **p < 0.01 vs. the untreated control cells

DOX increases the activity of caspase-3/7 [11, 17, 25], which plays an essential role in the apoptotic processes [34]. In a preliminary experiment, we determined the degree of caspase-3/7 activation in response to various concentrations (0.1–1 μM) of DOX in H9c2 cells (Fig. 1). These concentrations were chosen based on a pharmacokinetic observation in the human physiological context [35], in which DOX at an interstitial concentration of 0.1 μM or higher induces cytotoxicity in the heart. DOX significantly increased the caspase-3/7 activity after incubation for 4 h at a concentration of 1 μM (*p* < 0.05) and for 8 h at concentrations of 0.3 μM or higher (*p* < 0.01). Therefore, we used 1 μM DOX, which can activate caspases in an early stage of incubation, for subsequent experiments.

**Fig. 2:**
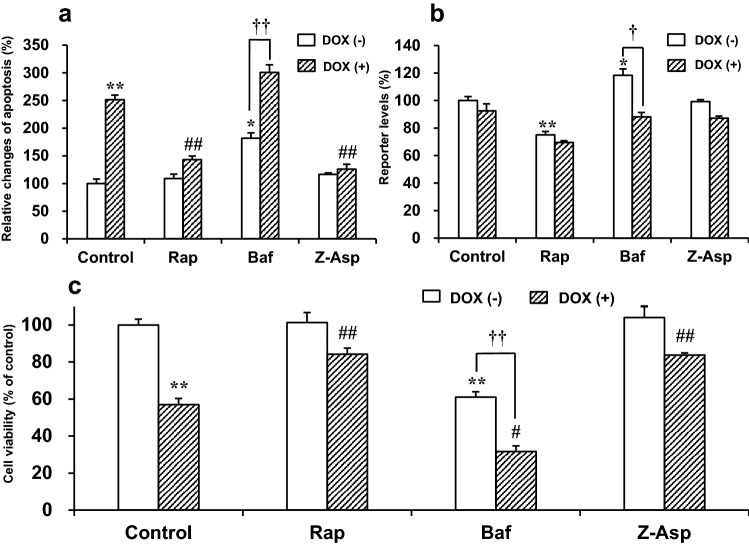
Effects of Rap, Baf, or Z-Asp on doxorubicin (DOX)-induced changes in **a** apoptosis, **b** autophagy, and **c** cell viability in H9c2 cells. The cells were pretreated with 50 nM Rap, 10 nM Baf, or 100 μM Z-Asp for 1 h and then incubated with 1 μM DOX. Each parameter was assessed after incubating the cells for 8 h (**a**, **b**) or 18 h (**c**) with DOX. Apoptosis and autophagy were detected based on the luminescent signal indicating the Annexin V-positive cells and based on the level of LC3 HiBiT reporter activity. The relative cell viability was determined through the luminescence assay based on adenosine triphosphate quantification (CellTiter-Glo® 2.0 assay). Each value was expressed as a percentage relative to that from DOX-untreated control cells. Data are presented as the mean ± SEM of three samples. Rap: rapamycin. Baf: bafilomycin A1. Z-Asp: Z-Asp-CH_2_-DCB. DOX: doxorubicin. *p < 0.05, **p < 0.01 vs. the DOX-untreated control group. # *p* < 0.05, ## *p* < 0.01 vs. the DOX-treated control group. †*p* < 0.05, ††*p* < 0.01 when compared between the Baf-untreated and -treated groups

Figure 2 shows the effects of Rap (an autophagy inducer), Baf (an autophagy inhibitor), and Z-Asp (a broad caspase inhibitor) on apoptosis, autophagy, and cell viability in DOX-treated and -untreated H9c2 cells. In the absence of Rap, Baf, or Z-Asp (i.e., control group), DOX (1 μM) significantly induced cellular apoptosis and viability reduction after incubation for 8 h and 18 h, respectively. No reduction in viability was observed before the onset of apoptosis induction. The apoptotic level and cell viability of DOX-treated cells were 251.4% (*p* < 0.01) and 57.0% (*p* < 0.01), respectively, of those of DOX-untreated control cells (Fig. 2a, c). These results suggest that DOX induces the activation of caspase-3/7 and thereby apoptosis and subsequent death in H9c2 cells.

As shown in Fig. 2b, the luminescent signal produced in the autophagy assay was decreased by Rap (*p* < 0.01) but was increased by Baf (*p* < 0.05) in DOX-untreated cells, indicating the promotive effect of Rap and the inhibitory effect of Baf on autophagy. DOX (1 μM) itself did not modify the basal level of autophagy in H9c2 cells (“control group” in Fig. 2b). Both cellular apoptosis (*p* < 0.01; Fig. 2a) and viability reduction (*p* < 0.01; Fig. 2c) induced by DOX were markedly attenuated by incubation with Rap prior to DOX exposure. Similarly, the pretreatment with Z-Asp effectively prevented these DOX-induced cellular events (*p* < 0.01; Fig. 2a, c). The treatment with Baf produced significant effects under basal condition (absence of DOX); it promoted apoptosis induction (*p* < 0.05; Fig. 2a) and cell death (*p* < 0.01; Fig. 2c), with inhibition of autophagy. When the Baf treatment was followed by exposure to DOX, it led to a further reduction in cell viability (*p* < 0.05; Fig. 2c). Baf also tended to enhance apoptosis after DOX exposure, although the level was statistically insignificant (*p* > 0.05) when compared to the DOX-treated control cells (Fig. 2a). The autophagy level, which had been decreased by Baf, returned to the basal level after DOX exposure (*p* < 0.05), possibly because of the ability of DOX to reduce LC3 levels, which had been increased by Baf [18]. Neither Rap, Baf, nor Z-Asp interfered with the assays for apoptosis, autophagy, and cell viability (data not shown). These results indicate that the induction of autophagy prior to DOX administration can protect cardiomyocytes from DOX-induced apoptosis and viability reduction.

### Transfection of Pdcd1 Expression Vector and Detection of Autophagy

**Fig. 3:**
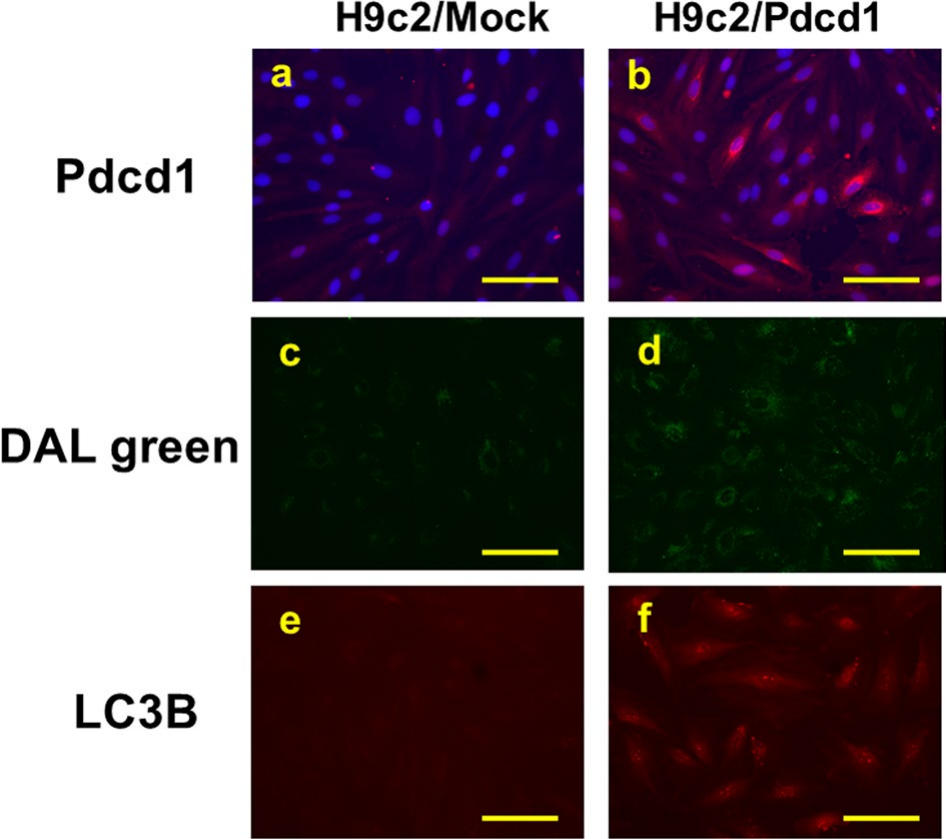
Detection of Pdcd1 expression and autophagy in Pdcd1-overexpressing H9c2 (H9c2/Pdcd1) cells and negative control (H9c2/mock) cells. Pdcd1-overexpressing cells were established by transfecting H9c2 cells with Pdcd1-encoding plasmid DNA. After 24 h of transfection, the Pdcd1 protein expression (red fluorescence) was determined through immunofluorescence analysis in H9c2/mock (**a**) or H9c2/Pdcd1 cells (**b**). Autophagy was detected through staining with DAL Green, an autolysosome detection reagent (green fluorescence), and by the expression of LC3B, an autophagy marker, using immunofluorescence analysis in H9c2/mock (**c**, **e**) and H9c2/Pdcd1 cells (**d**, **f**). Magnification =  × 400. Scale bar = 100 μm

We transfected H9c2 cells with Pdcd1-encoding plasmid DNA to establish Pdcd1-overexpressing H9c2 (H9c2/Pdcd1) cells and confirmed the overexpression by an immunofluorescence assay. To determine whether autophagy was induced in H9c2/Pdcd1 cells, we visually assessed the appearance of autolysosomes through fluorescence analysis and detected LC3B expression through immunofluorescence analysis (Fig. 3). In addition, the overexpression of Pdcd1 in H9c2/Pdcd1 cells was confirmed through western blotting (Fig. S1). The results showed that both autolysosome and LC3B levels were apparently high in H9c2/Pdcd1 cells compared to the negative control (H9c2/mock) cells, suggesting that Pdcd1 overexpression promotes autophagy induction in H9c2 cells.

### DOX-Induced Apoptosis and Caspase-3/7 Activation in H9c2/Pdcd1 Cells

**Fig. 4:**
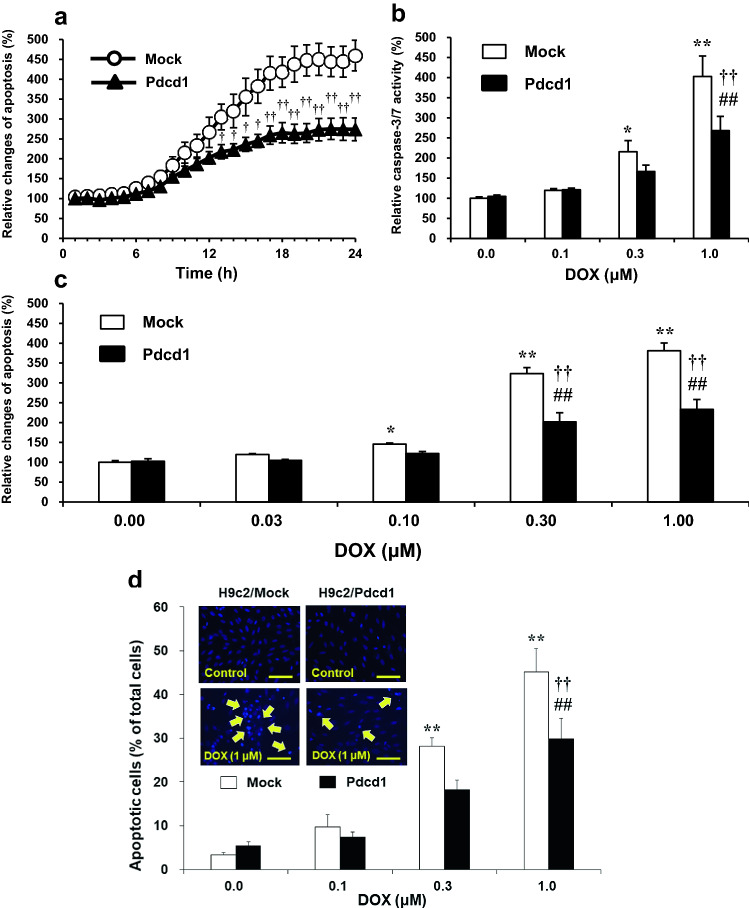
Effects of Pdcd1 overexpression on doxorubicin (DOX)-induced apoptosis and caspase-3/7 activation in H9c2 cells. **a** After the cells were incubated with 1 μM DOX for various time periods (1–24 h), apoptosis was evaluated through a luciferase assay indicating the increase in Annexin V-positive cells. **b** Activity of caspase-3/7, an executor enzyme of apoptosis, was assessed using a luciferase assay in cells incubated with 1 μM DOX for 8 h; the values were expressed as the percentage of DOX-untreated H9c2/mock control cells. **c** Apoptosis levels in response to various concentrations of DOX (0.03–1 μM). The apoptosis was determined after a 24 h incubation with DOX, using the Annexin V luciferase assay. Each value was expressed as a percentage of that in H9c2/mock control cells. **d** Nuclear morphological observations and quantification of the apoptotic cells stained with H33342 after a 24 h incubation with various concentrations of DOX (0.1–1 μM). The yellow arrows indicate a typical feature of apoptotic cells. Magnification =  × 200. Scale bar = 50 μm. Data are presented as the mean ± SEM of three samples. *p < 0.05, **p < 0.01 vs. the DOX-untreated mock (control) cells. ## *p* < 0.01 vs. the DOX-untreated Pdcd1 over-expressed cells. †*p* < 0.05, ††*p* < 0.01 vs. the corresponding DOX-treated mock cells

Figure 4 shows the degree of DOX-induced apoptosis and caspase-3/7 activation in H9c2/mock and H9c2/Pdcd1 cells. We first evaluated the time-course changes in DOX (1 μM)-induced apoptosis using a luminescence assay. Apoptosis increased with prolonged DOX treatment in both H9c2/Pdcd1 and H9c2/mock cells but was significantly reduced in the former than in the latter cells 12 to 24 h after DOX incubation (*p* < 0.05; Fig. 4a). We next examined the effects of various DOX concentrations on the caspase-3/7 activity and apoptosis in H9c2/mock and H9c2/Pdcd1 cells. The caspase activity was measured after incubation for 8 h with DOX (Fig. 4b), and apoptosis was detected by a luminescence assay (Fig. 4c) or nuclear morphological observation (Fig. 4d) after incubation for 24 h with DOX. DOX caused caspase-3/7 activation and apoptosis induction at similar concentrations in both cell types. These alterations induced by DOX (1 μM) were markedly attenuated in H9c2/Pdcd1 cells compared to H9c2/mock cells (*p* < 0.01; Fig. 4c, d). These results suggest that Pdcd1 overexpression can prevent DOX-induced caspase activation and subsequent apoptosis in H9c2 cells.

### Pathways of Autophagy and Apoptosis in H9c2/Pdcd1 Cells

**Fig. 5:**
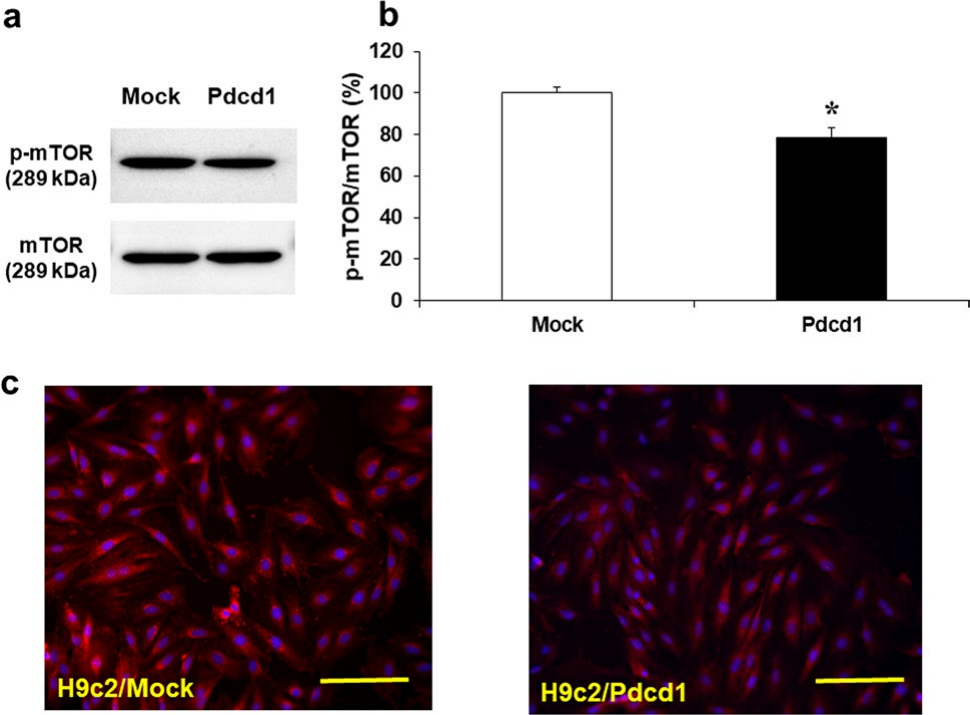
Expression of phosphorylated mTOR (p-mTOR) and mTOR proteins in H9c2/mock and H9c2/Pdcd1 cells under DOX-untreated basal condition. **a** The expression of p-mTOR and mTOR in both cell types were evaluated through western blotting. **b** Quantitative levels of p-mTOR/mTOR protein expression. Data are presented as the mean ± SEM of three samples. *p < 0.05 vs. the mock cells. **c** Immunofluorescence detection of p-mTOR expression in H9c2/mock and H9c2/Pdcd1 cells. Magnification =  × 200. Scale bar = 50 μm

In order to elucidate the mechanisms underlying autophagy induction in H9c2/Pdcd1 cells, we detected the protein expression of mTOR, a major negative regulator of autophagy, and its active form, p-mTOR, in DOX-untreated cells using western blotting (Fig. 5a, b). The expression ratio of p-mTOR to mTOR in H9c2/Pdcd1 cells decreased to 78.4% (*p* < 0.05) compared to that in H9c2/mock cells. The results of the immunofluorescence study also revealed that p-mTOR expression was more extensively reduced in H9c2/Pdcd1 cells than in H9c2/mock cells (Fig. 5c).

**Fig. 6:**
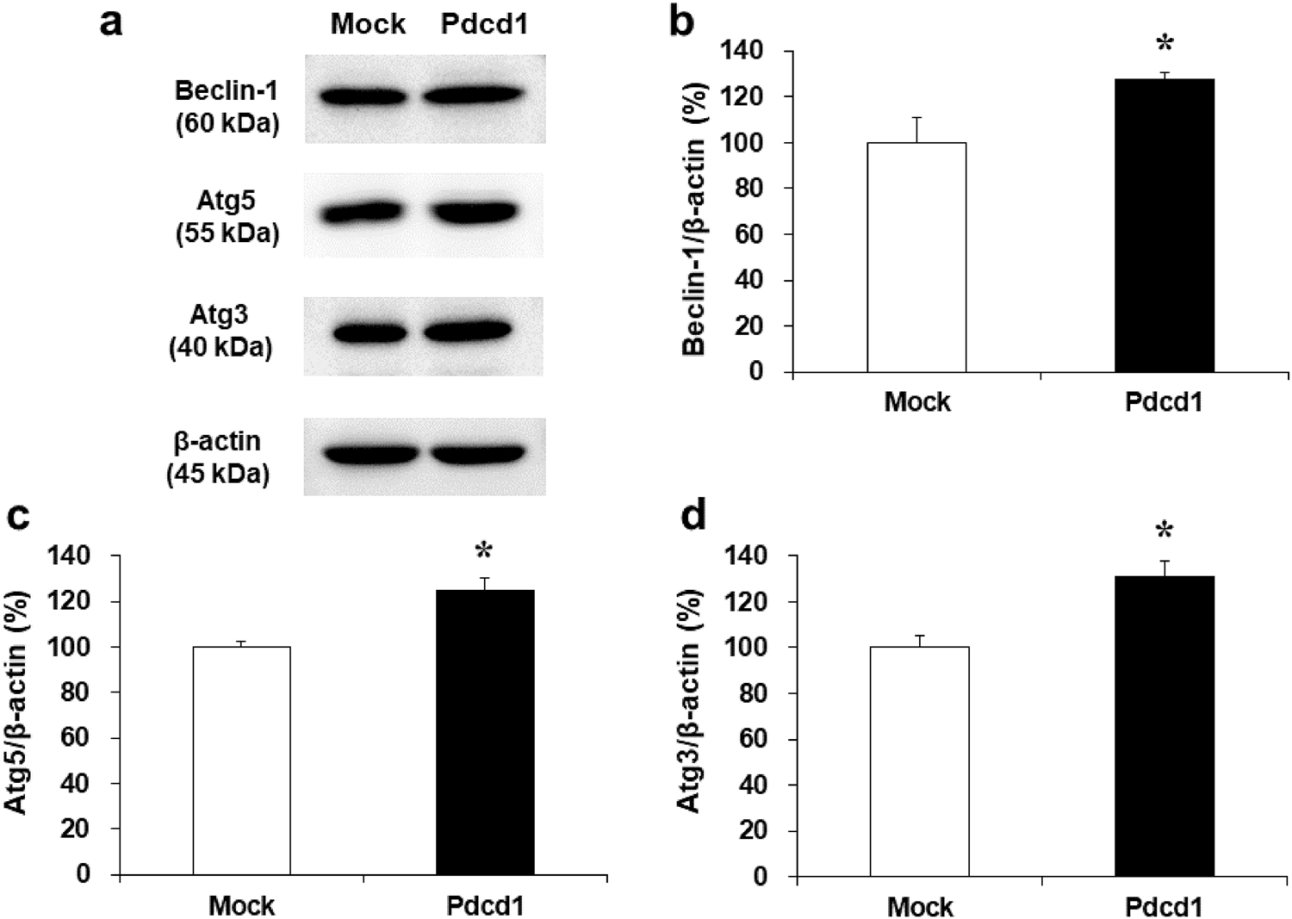
Expression of Beclin-1, Atg5, and Atg3 proteins in H9c2/mock and H9c2/Pdcd1 cells under DOX-untreated basal condition. **a** The expression of Beclin-1, Atg5, and Atg3 in both cell types were evaluated using western blotting, with β-actin as the loading control. **b**–**d** Quantitative levels of protein expression. Data are presented as the mean ± SEM of three samples. *p < 0.05 vs. the mock cells

**Fig. 7:**
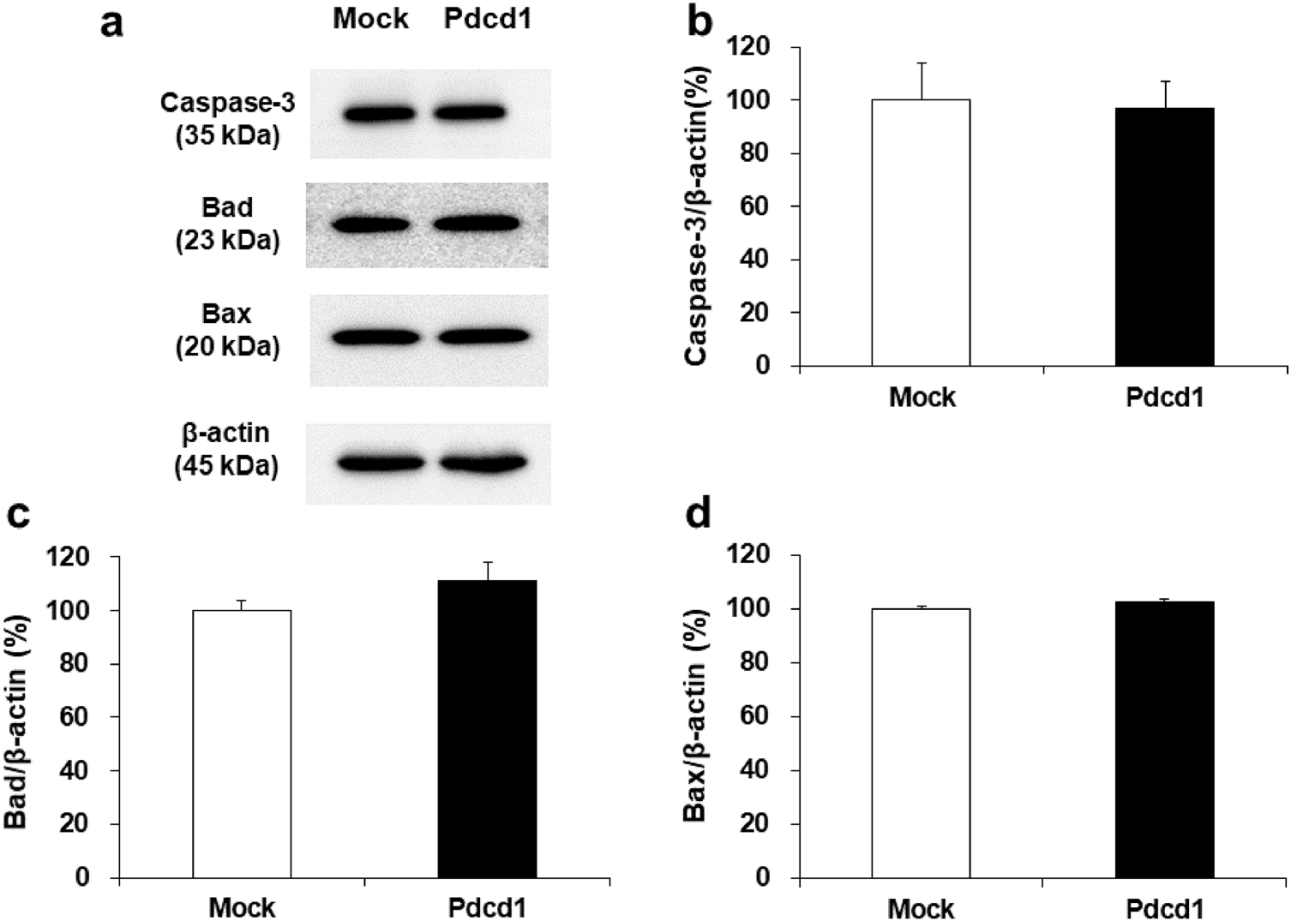
Expression levels of caspase-3, Bad, and Bax proteins in H9c2/mock and H9c2/Pdcd1 cells under DOX-untreated basal condition. **a** The expression of the caspase-3, Bad, and Bax proteins in both cell types were evaluated using western blotting, with β-actin as the loading control. **b**–**d** Quantitative levels of protein expression. Data are presented as the mean ± SEM of three samples

We further evaluated the expression of the downstream proteins of mTOR signaling in the absence of DOX (Fig. 6). The expression of Beclin-1, Atg5, and Atg3 in H9c2/Pdcd1 cells increased to 127.8% (*p* < 0.05; Fig. 6b), 124.9% (*p* < 0.05; Fig. 6c), and 131.0% (*p* < 0.05; Fig. 6d), respectively, compared to H9c2/mock cells. Moreover, to determine whether Pdcd1 can directly regulate the expression of key proteins in the apoptosis pathway, the expression of caspase-3, Bad, and Bax in DOX-untreated cells was assessed by western blotting (Fig. 7). No significant difference was observed in the expression of these proteins between H9c2/Pdcd1 and H9c2/mock cells. These results suggest that Pdcd1 signaling does not have a prominent direct effect on the apoptosis pathway, but activates autophagy pathway through the inhibition of mTOR expression in H9c2 cells.

### Effects of Rap and Baf on Autophagy Induction in H9c2/Pdcd1 Cells

**Fig. 8:**
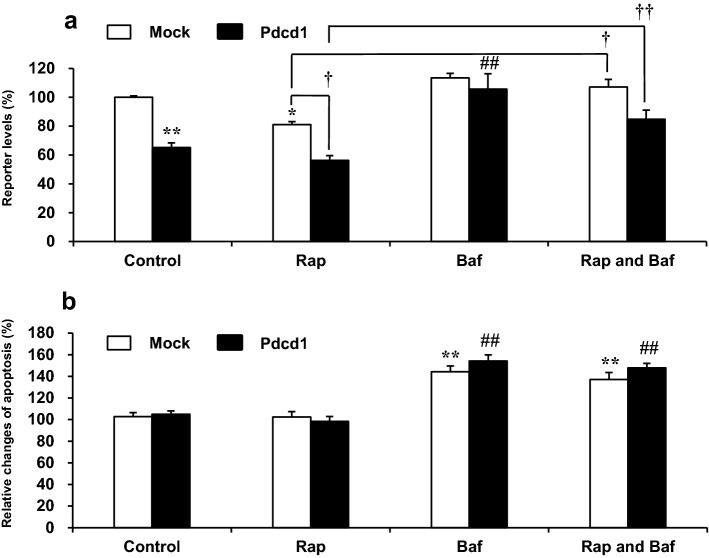
Changes in the basal levels of autophagy and apoptosis after incubation for 6 h with Rap, Baf, or their combination in H9c2/mock and H9c2/Pdcd1 cells under DOX-untreated basal condition. **a** Autophagy was evaluated using the autophagy LC3 HiBiT reporter assay. **b** Apoptosis was determined using a luciferase assay indicating increase in Annexin V-positive cells. Each value is expressed as a percentage relative to that in H9c2/mock control cells. Data are presented as the mean ± SEM of three samples. *p < 0.05, **p < 0.01 vs. the control group in H9c2/mock cells; ## *p* < 0.01 vs. the control group in H9c2/Pdcd1 cells; †*p* < 0.05, ††*p* < 0.01 vs. the corresponding Rap-treated group

To further confirm the induction of autophagy by Pdcd1 overexpression, the effects of Rap, Baf, and their combination on autophagy levels of DOX-untreated H9c2/Pdcd1 cells were determined using the LC3 HiBiT reporter assay (Fig. 8a). In the absence of Rap, Baf, or both (control group), the LC3 reporter levels in H9c2/Pdcd1 cells decreased to 65.2% of that in H9c2/mock cells (*p* < 0.01), indicating the induction of autophagy in H9c2/Pdcd1 cells. Rap decreased the LC3 reporter levels in both H9c2/mock and H9c2/Pdcd1 cells; in particular, the decrease in the level caused by Pdcd1 overexpression was additively enhanced by Rap (*p* < 0.05). Conversely, Baf almost completely restored the LC3 reporter levels, which were decreased by Pdcd1 overexpression, and inhibited the enhanced decrease in LC3 reporter levels observed in the Rap-treated H9c2/Pdcd1 cells. These results strongly suggest that Pdcd1 signaling activates the autophagy pathway in H9c2 cardiomyocytes.

In addition, we assessed whether Rap or Baf modified the spontaneous basal level of apoptosis in H9c2/mock and H9c2/Pdcd1 cells under DOX-untreated conditions (Fig. 8b). No difference in the luminescence intensity of Annexin V between H9c2/mock and H9c2/Pdcd1 cells was observed in the absence of Rap, Baf, or both (control group), indicating that Pdcd1 signaling does not have a prominent effect on the basal apoptosis of H9c2 cells. Similarly, no significant change in apoptosis was observed, even when autophagy was induced by Rap. In contrast, Baf induced apoptosis in both H9c2/mock and H9c2/Pdcd1 cells, in association with the inhibition of autophagy. Apoptosis induction by Baf was observed in these two cells to a similar degree, regardless of the presence or absence of Rap. It is, therefore, possible that autophagy activation by Pdcd1 or Rap does not practically affect the basal apoptosis of H9c2 cells, whereas autophagy inhibition by Baf promotes it.

### Effect of Pdcd1 Overexpression on DOX-Induced Apoptosis in Cancer Cells

**Fig. 9:**
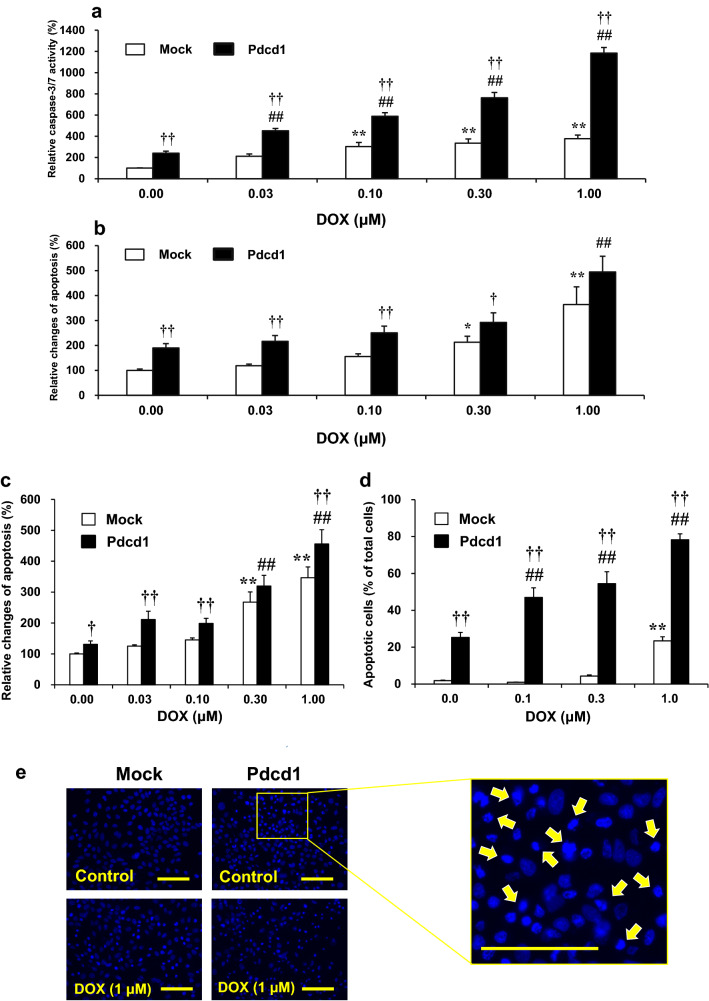
Effects of Pdcd1 overexpression on doxorubicin (DOX)-induced apoptosis in human cancer cell lines, K562 (human erythroleukemia cells) and MCF-7 (human breast cancer cells). **a** Caspase-3/7 activity in the control (mock) and Pdcd1-overexpressing (Pdcd1) K562 cells was measured using a luciferase assay after incubation with 1 μM DOX for 8 h. The values were expressed as a percentage relative to that in DOX-untreated mock cells. **b**, **c** The apoptosis was assessed after a 24 h incubation with various concentrations of DOX (0.03–1 μM) using the Annexin V luciferase assay in K562 (**b**) and MCF-7 cells (**c**). Each value was expressed as a percentage relative to that in DOX-untreated control (mock) cells. **d** Quantification of apoptotic cells after a 24 h incubation with various concentrations of DOX (0.1–1 μM) in the control (mock) and Pdcd1-overexpressing MCF-7 cells. Nuclear morphological observations were performed after staining with H33342. **e** Control (mock) and Pdcd1-overexpressing (Pdcd1) MCF-7 cells stained with H33342 after a 24 h incubation with 1 μM DOX. Yellow arrows indicate a typical feature of apoptotic cells. Magnification =  × 200. Scale bar = 50 μm. Data are presented as the mean ± SEM of three samples. *p < 0.05, **p < 0.01 vs. the DOX-untreated mock (control) cells; ## *p* < 0.01 vs. the DOX-untreated Pdcd1-overexpressing cells; †*p* < 0.05, ††*p* < 0.01 vs. the corresponding DOX-treated mock cells

To determine the role of Pdcd1 in DOX-induced apoptosis of cancer cells, K562 and MCF-7 cells were transfected with Pdcd1 overexpression plasmid (Fig. 9). In the Pdcd1-overexpressing K562 cancer cells, the basal levels of caspase-3/7 activity and apoptosis (as determined by the luminescence intensity of Annexin V) were considerably higher, even in the absence of DOX; these levels were 240.6% (*p* < 0.01) and 190.0% (*p* < 0.01), respectively, of those in the control (mock) cells. DOX-induced concentration-dependent increases in caspase-3/7 activity and apoptosis in mock cells (Fig. 9a, b). DOX-induced apoptosis was additively enhanced in Pdcd1-overexpressing cells (Fig. 9b).

Because MCF-7 cancer cells are deficient in caspase-3 [36], we did not measure the caspase-3/7 activity in these cells. Alternatively, in addition to K562 cells, Pdcd1-overexpressing MCF-7 cells also showed the induction of apoptosis, as determined by the luminescence intensity of Annexin V (Fig. 9c) and morphological observation of nuclei (Fig. 9d, e). DOX enhanced apoptosis induction in Pdcd1-overexpressing cells; the enhancement was especially remarkable when the degree of apoptosis was assessed by nuclear morphological changes (i.e., chromatin condensation followed by partition into multiple bodies) (Fig. 9d). These results suggest that in K562 and MCF-7 cells, Pdcd1 signaling can promote apoptosis induction as well as enhance DOX-induced apoptosis. Therefore, the role of Pdcd1 in regulating apoptosis in cancer cells is in contrast to that in H9c2 cardiomyocytes. In addition, there was no induction of autophagy in cancer cells (K562 and MCF-7), regardless of Pdcd1 overexpression (data not shown).

## Discussion

DOX cardiotoxicity is mediated by at least two major pathways of apoptosis and autophagy [4, 8–12]. Apoptosis has been demonstrated to be an important process involved in the mechanisms of DOX-induced cardiomyocyte death and cardiotoxicity. In fact, DOX-induced cardiomyocyte apoptosis is mediated by the intrinsic and extrinsic apoptotic pathways [37, 38]. The results of the present study also showed that DOX promoted apoptosis and reduced the viability of H9c2 cardiomyocytes (Fig. 2), both of which were accompanied by the early activation of pro-apoptotic enzymes caspase-3/7 (Fig. 1). Moreover, caspase inhibition with Z-Asp mostly inhibited both apoptosis and viability reduction in DOX-treated H9c2 cells (Fig. 2). These facts suggest that DOX-induced cardiomyocyte death is mainly mediated by the activation of the apoptotic pathway, in agreement with previous findings [9, 16–19].

Autophagy has dual functions depending on cellular conditions. Under physiological conditions, as it eliminates damaged or unwanted proteins and organelles from the cells and, thus, is essential for optimal cellular function and survival. Under pathological conditions, autophagy may be induced to protect cells from various stress stimuli or, alternatively, to contribute to cell death [39]. Recently, the role of autophagy in DOX-induced cardiotoxicity has been explored in many studies; however, its role is complex and not fully understood [39, 40]. According to the review by Koleini and Kardami [8], boosting autophagy prior to the administration of DOX can reduce DOX-induced cardiotoxicity. For example, cardiomyocytes were rescued from DOX-induced toxicity when autophagy was induced by Rap before the DOX treatment [17, 18]. Similar cardioprotection has been shown in animals, in which autophagy was induced by nutrient starvation [16]. In contrast, autophagy signaling following DOX administration likely contributes to DOX-induced toxicity [8]. We observed that DOX-induced apoptosis and death were inhibited by pretreatment with Rap, and that the death was accelerated by pretreatment with Baf (Fig. 2). The activated autophagy pathway can prevent the DOX-induced increase in caspase activity in H9c2 cells [17]. Therefore, autophagy induction prior to DOX administration may be an important event for the inhibition of caspase activity and hence protection from DOX-induced cardiomyocyte apoptosis and toxicity.

We previously found that the plasma mRNA levels of Pdcd1 before DOX administration were significantly and positively correlated with the severity of DOX-induced cardiotoxicity in mice [25]. Interestingly, the small interfering RNA-mediated knockdown of Pdcd1 potentiated DOX-induced apoptosis in H9c2 cells, suggesting that Pdcd1 serves as a protective molecule against DOX-induced cardiomyocyte apoptosis [25]. In the present study, the Pdcd1-overexpressing H9c2 cells showed the attenuation of both caspase-3/7 activation and DOX-induced apoptosis (Fig. 4), whereas these cells showed no change in the expression of apoptotic proteins, caspase-3, Bad, and Bax under basal conditions without DOX (Fig. 7). These results indicate that Pdcd1 signaling can inhibit DOX-induced apoptosis without directly affecting the apoptosis pathway in cardiomyocytes.

**Fig. 10:**
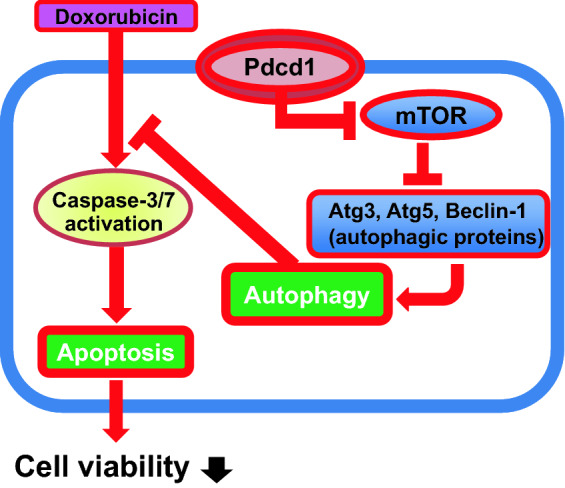
Schematic representation of the molecular mechanisms by which Pdcd1 overexpression plays a protective role against DOX-induced apoptosis and cell viability reduction in the cardiomyocyte H9c2 cells. Pdcd1-mediated signaling inhibits the expression of mTOR; and therefore, increases the expression of its downstream proteins, Atg3, Atg5, and Beclin-1, leading to the activation of autophagy, which inhibits the DOX-induced caspase activation and subsequent apoptosis. Therefore, Pdcd1 could be an effective molecule for cardioprotection from DOX-induced toxicity

One of the most remarkable findings of this study is that Pdcd1-overexpressing H9c2 cells also showed apparent induction of autophagy, as evidenced by increases in autolysosome formation and LC3B expression (Fig. 3). This autophagy induction could be attributed to the reduced activity of mTOR for the following three reasons. First, Pdcd1 overexpression decreased the expression of p-mTOR (Fig. 5). Second, it also increased the expression of Atg3, Atg5, and Beclin-1, the major downstream effectors of mTOR (Fig. 6). Finally, autophagy induction was additively, but not synergistically, enhanced by the application of Rap (an mTOR inhibitor) to Pdcd1-overexpressing H9c2 cells (Fig. 8a). The increased activities of Atg3, Atg5, and Beclin-1 in the autophagy pathway can trigger caspase activity inhibition in the apoptosis pathway [41, 42]. In accordance with this fact, Pdcd1 overexpression inhibited the activation of caspase-3/7 following exposure to DOX (Fig. 4b). Taken together, it seems that Pdcd1 signaling inhibits mTOR expression and causes the secondary activation of the autophagy pathway, leading to the prevention of DOX-induced caspase activation and subsequent apoptosis (Fig. 10). Interestingly, the genetic or pharmacological inhibition of mTOR can also prevent cardiac diseases, including cardiac remodeling and heart failure, in response to pressure overload and chronic myocardial infarction [43].

On the other hand, mTOR plays a pivotal role in the regulation of the immune system, mediating T-cell activation [44], and Pdcd1 is an important immune checkpoint target in T cells. Celada et al. showed that the upregulation of Pdcd1 was negatively correlated with mTOR activation in T cells in patients with sarcoidosis [45]. Pdcd1 also contributes to immune responses in the heart [46]. It has been reported that the deletion of Pdcd1 causes autoimmune myocarditis [47], and that Pdcd1 protects against inflammation and myocyte damage in the heart [48]. However, the detailed relationship between mTOR and Pdcd1 in the heart tissue remains obscure.

Another remarkable finding of this study is that the overexpression of Pdcd1 in cancer cells promoted apoptosis induction, which was further enhanced by DOX (Fig. 9b–e). These alterations in K562 cancer cells were accompanied by an increase in the caspase-3/7 activity (Fig. 9a), although the enzyme was deficient in MCF-7 cancer cells. Thus, the role of Pdcd1 in cancer cells could be different from that in cardiomyocytes. According to tumor pathological studies, Pdcd1 expression is very high in T cells in the tumor microenvironment in cancer patients [49], and it is correlated with tumor cell malignancy [50]. In vitro overexpression of Pdcd1 could induce apoptosis in the human SKOV3 ovarian cancer cell line and significantly strengthened cisplatin-induced apoptosis [51]. It is possible that Pdcd1 signaling promotes apoptosis in cancer cells, while it has anti-apoptotic properties in cardiomyocytes. To the best of our knowledge, this is the first report to demonstrate the cardioprotective role of Pdcd1 mediated by the autophagy pathway. Pdcd1 may be a valuable target for more effective and safe DOX chemotherapy. However, the apoptotic mechanisms of Pdcd1 overexpression in cancer cells remain unclear. Further studies are needed to determine the detailed pathways and mechanisms underlying the apoptotic role of Pdcd1 in cancer cells.

In conclusions, the results of the present study revealed that the role of Pdcd1 in DOX-induced apoptosis is opposite between cardiomyocytes and cancer cells. The overexpression of Pdcd1 in cardiomyocytes attenuated DOX-induced cellular apoptosis and death. The underlying mechanism of the beneficial role of Pdcd1 is thought to involve the induction of autophagy, resulting from the inhibition of mTOR and subsequent induction of downstream autophagic proteins such as Beclin-1, Atg3, and Atg5 (Fig. 10). In contrast, Pdcd1 overexpression in cancer cells promotes the induction of apoptosis, which is further enhanced by DOX. Therefore, Pdcd1 could be a critical molecule for both the prevention of DOX cardiotoxicity and effective chemotherapy with DOX.

## Supplementary Information

Below is the link to the electronic supplementary material.

Fig. S1 Expression of Pdcd1 protein in H9c2/mock and H9c2/Pdcd1 cells. (a) The expression of Pdcd1 in both cell types were evaluated through western blotting, using β-actin as the loading control. (b) Quantitative levels of protein expression. Data are presented as the mean ± SEM of three samples. ** p <0.01 vs. the mock cells (TIF 323 kb)

## Data Availability

The datasets generated during and/or analyzed during the current study are available from the corresponding author on reasonable request.

## Abbreviations

DOX: Doxorubicin
Pdcd1: Programmed cell death 1
mTOR: Mammalian target of rapamycin
Rap: Rapamycin
Baf: Bafilomycin A1
Z-Asp: Z-Asp-CH_2_-DCB
LC3B: Light chain 3B
Atg3: Autophagy gene 3
Atg5: Autophagy gene 5
Bad: Bcl-2-associated agonist of cell death protein
Bax: Bcl-2-associated X protein
PBS: Phosphate-buffered saline
